# Inpatient outcomes of upper cervical fractures in the elderly: a retrospective analysis of 268 cases

**DOI:** 10.1186/s12877-025-05862-4

**Published:** 2025-04-09

**Authors:** Nicolas H. von der Höh, Jonathan Keuchel, Phillip Pieroh, Ulrich Josef Albert Spiegl, Georg Osterhoff, Christoph-Eckhard Heyde

**Affiliations:** 1 Department for Orthopaedics, Trauma Surgery and Plastic Surgery, University Hospital Leipzig, Liebigstr. 18, Leipzig, 04103 Germany; 2 Department of Anesthesiology and Intensive Care Medicine, Heart Center Leipzig, Leipzig, Germany; 3 Department for Orthopaedics, Trauma Surgery and Plastic Surgery, Munich Harlaching Hospital, Munich, Germany

**Keywords:** Cervical spine fracture, Elderly patients, Upper cervical injuries, Surgical vs conservative treatment, In-hospital mortality, Odontoid / atlas fracture

## Abstract

**Background:**

The incidence of upper cervical fractures in elderly individuals is increasing, necessitating enhanced treatment approaches.

**Method:**

A retrospective study of 268 elderly patients with upper cervical fractures was conducted to assess inpatient outcomes aged 75 and older with atlas and/or axis fractures. Patient risk was evaluated using the ASA score and Charlson comorbidity index (CCI). In-hospital mortality and functional outcomes were assessed, with fracture treatment strategies following AO principles.

**Results:**

Patients with C1, C2 or combined fractures did not differ in age, CCI, Barthel score or length of hospital stay (*p* > 0.05). C2 fractures were predominant, and the majority of patients suffered at least from a severe general disease (ASA ≥ 3). Comparing operatively and conservatively treated patients, regardless of fracture localization, revealed no significant differences in mortality, both overall and time-related. Surgical patients experienced a higher frequency of general complications, notably dyspnea. The overall mortality rate was 14.9%, with 15.7% in the nonoperative group and 14.4% in the surgical group (*p* = 0.8628). The overall rate of general complications was 51.4% (*n* = 51) in the nonoperative group and 71.9% (*n* = 110) in the operatively treated group. Anterior fixation procedures showed significantly higher rates of pneumonia and respiratory complications, while mortality and other complications did not differ significantly between posterior and anterior surgical approaches.

**Conclusion:**

The in-hospital mortality and morbidity of elderly patients with upper cervical fractures are high but do not significantly differ between operatively and nonoperatively treated patients. The complexity of the geriatric patient population highlights the need for peri- and postinpatient geriatric complex treatment, emphasizing the importance of establishing geriatric-specialized care structures.

## Background

Upper cervical fractures after falls are among the most common fractures in geriatric patients. Underlying causes are altered posture with a ventrally shifted center of gravity, osteoporosis and sarcopenia, and a deteriorated sense of balance [1, 2]. In addition, there are several comorbidities that influence the risk of falls as well as outcomes [3, 4]. Osteoporosis is a particular risk factor [5–8], and in connection with poor bone quality, even low-energy trauma leads to serious injury patterns [3, 9]. In the Western world, we are currently in the midst of a rapid increase in the aging population. The peak is expected to be reached at the beginning of the retirement age of the largest birth cohort in 1964. It is estimated that by 2050, the proportion of people aged 80 or older in the European Union will more than double, reaching 11.4% [10]. As one of the effects, odontoid fractures in patients older than 70 years of age show a significantly increasing incidence in the twenty-first century compared with those under 70 years of age [10]. In this regard, the frequently multimorbid patient population plays an overriding role in clinical care. In addition to age, common secondary diseases in geriatric patients in the literature are diabetes mellitus, osteoporosis, and cardiac and pulmonary diseases [5, 11, 12]. Not least because of the high age, a general mortality risk is also found in the group of patients over 75 years of age during inpatient treatment [6, 13]. For odontoid fractures, different intrahospital mortality rates are found in the literature, with most studies focusing on a study period beyond the inpatient stay, and it is not always clear whether patients died in the inpatient stay or afterwards. In these studies, the 1-year mortality for conservatively treated patients ranged from 0 to 14%, and for operatively treated patients, it ranged from 8 to 37.5% [11, 14–19]. A study that purely examined intrahospital outcomes in terms of mortality and morbidity does not exist in the literature. While treatment strategies and care in younger patients follow clear guidelines, we still face a major challenge with regard to therapeutic decisions regarding upper cervical spine fractures in geriatric patients.

Particularly in geriatric patients, we register a high rate of nonunion (conservative as well as operative) due to poor bone quality, regional biomechanical conditions and insufficient blood supply [20].

Thus, in addition to radiological criteria, neurological deficits and instability, bone quality and especially patient age should be considered in the choice of therapy. Especially in elderly patients, the treatment strategy is highly dependent on various factors. An essential one, however, regards the general condition of the patient because in addition to the operative risk, the anesthesiologic risk increases with increasing age [21–23].

Iyer et al. constructed an operating algorithm for odontoid fractures based on the literature [24].

However, it is very important to obtain data on the strictly inpatient setting to be able to condition patients preoperatively, if necessary.

The purpose of this study was to compare the morbidity and mortality of patients after operative and nonoperative treatment during the inpatient stay. The data collected should help to provide a realistic assessment of the expected risks of treatment after odontoid fractures and C1/2 fractures.

## Methods

The present study was approved by the local ethics committee (Number 306/20-ek). Retrospectively, all patients aged ≥ 75 years admitted to a level-1 trauma center between December 2012 and April 2018 because of an atlas and/or axis fracture following a low-energy trauma were identified using the ICD-10 codes S12.0, S12.1, and S12.7.

Perioperative risk was evaluated with the ASA-Score (American Sociation of Anesthesiology) [5, 11, 12] and using the Charlson comorbidity index (CCI) to evaluate the patient´s morbidity [11, 14, 15]. Fractures of the atlas were classified according to Gehweiler [16], odontoid fractures by Anderson/D’Alonzo [17] and other C2 fractures.

The primary outcome was in-hospital mortality. Functional outcome was assessed using the Barthel Index, if available [18]. The length of hospital stay was determined for each patient. For each fracture group o*steoporosis was documented based on prior diagnosis in medical records, previous osteoporosis-related treatment, or available DXA/QCT measurements. Routine DXA screening was not performed for all patients.*

AO stability criteria were utilized to determine the treatment strategy. For C1 fractures, surgical indications were determined based on fracture type and stability. In particular, unstable type III and IV fractures, as described by Fiedler et al. [25] were considered for operative treatment.

Operative treatment included various fixation techniques. Posterior approaches consisted of C1-2 fusion, primarily performed using the Goel/Harms technique, or posterior C0-3 fusion in cases of craniocervical instability. Anterior procedures included odontoid double screw fixation for Anderson-D’Alonzo type II fractures, as well as three- and four-screw ventral screw fixation for unstable fractures and halo fixation. Nonoperative treatment was initiated in cases of stable fractures or when the patient declined surgery. Nonoperative therapy was performed by immobilization with a soft neck brace.

### Statistical analyses

Data are presented as the mean ± standard deviation (SD). Data were analysed regarding a Gaussian distribution using the Shapiro‒Wilk test. Due to the small number of operatively treated atlas fractures (*n* = 3), statistical comparisons between nonoperative and operative treatments were avoided. For the CCI of combined atlas and axis fractures, a Gaussian distribution was found, and a t test was used to compare nonoperative and operative therapy.

For the CCI and Barthel of axis fractures, Barthel of combined atlas and axis fractures and the data of length of hospital stay, a non-Gaussian distribution was found, and the Mann‒Whitney test was used to compare nonoperative and operative therapy. To compare nominal data of complications between nonoperative and operative therapy, Fisher´s exact test was used.

The level of significance was defined as *p* < 0.05. Analyses were performed with Graph Pad Prism software 8 (GraphPad software, La Jolla, USA). Additionally a Cox proportional-hazards regression analysis was performed to evaluate the association between patient characteristics, treatment methods, and in-hospital mortality. Independent variables included the ASA score (American Society of Anesthesiology), Charlson Comorbidity Index (CCI), treatment method (operative vs. conservative), and complications (e.g., sepsis, delirium, dyspnea). Time-to-event was defined as the duration from hospital admission to either death or discharge (censored). Hazard ratios (HRs) and 95% confidence intervals (CIs) were calculated. The analysis was conducted using Python with the lifelines package. Statistical significance was set at *p* < 0.05.

## Results

**Fig. 1:**
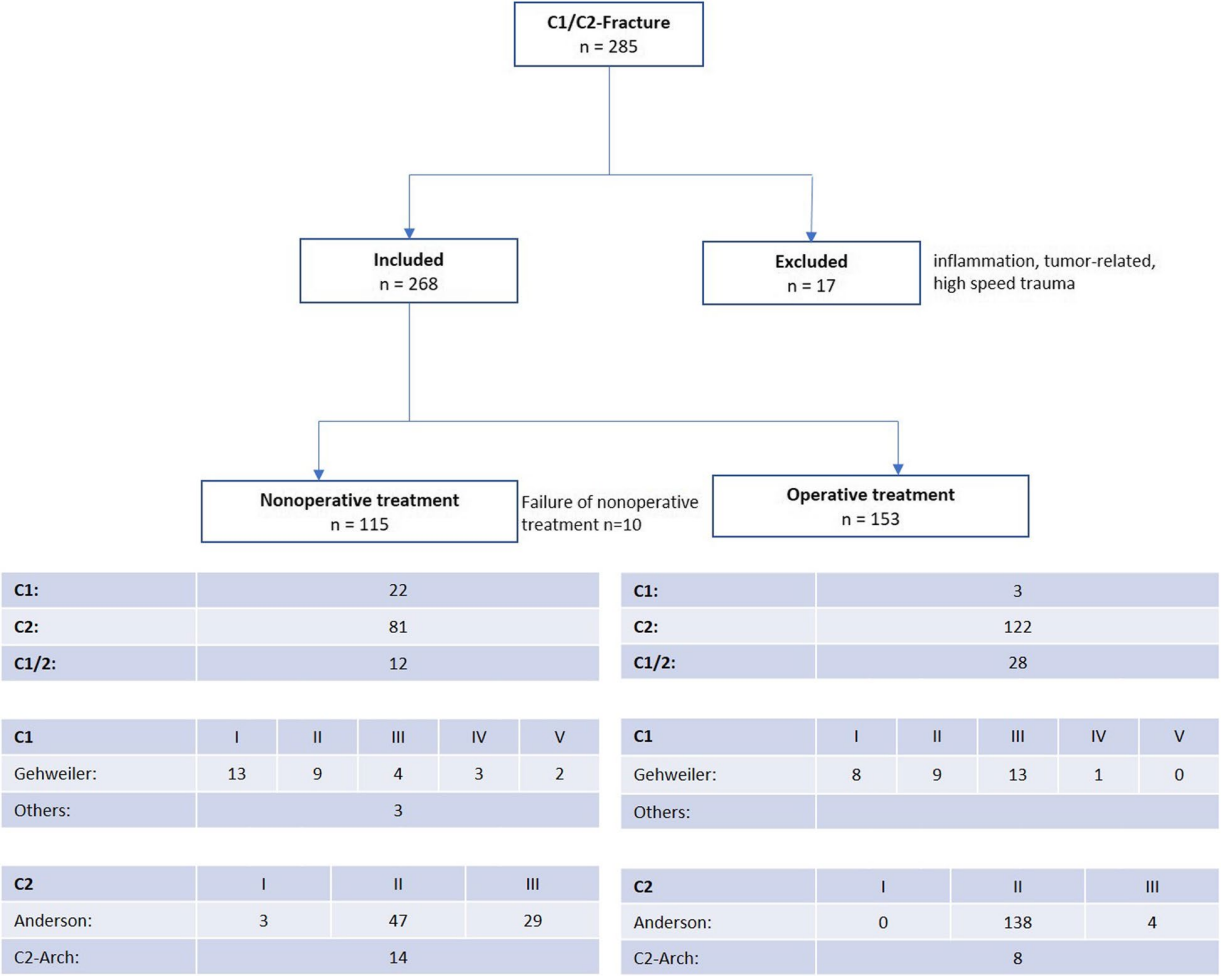
Patient flow chart: On the top, a total of 285 patients are mentioned, of which 268 have been included on the right

A total of 268 patients were included in the study. The mean age was 84.9 years. There were 115 patients in the nonoperative group and 153 patients in the operated group. Ten patients were converted from nonoperative to operative treatment, all of whom suffered from a C2 fracture classified as Anderson II (Fig. 1). The functional outcome was assessed using the Barthel Index, which was available for 207 out of the 268 patients.

Patients with C1, C2 or combined fractures did not differ in age, CCI, Barthel and length of hospital stay (Table 1, *p* > 0.05). C2 fractures were predominant, and the majority of patients suffered at least from a severe general disease (ASA ≥ 3).

**Table 1:** Patient baseline characteristics

	**C1 (*n* = 25)**	**C2 (*n* = 203)**	**C1/2 (*n* = 40)**
Sex (m:f)	10:25	60:143	12:27
Age	84.1 $\pm$ 5.9	84.8 $\pm$ 6.1	85.9 $\pm$ 5.5
ASA			
2	8	41	1
3	16	146	31
4	1	14	8
5	/	2	/
CCI	5.4 $\pm$ 1.6	6.1 $\pm$ 1.9	6.13 $\pm$ 1.7
Barthel (*n* =)	50.3 $\pm$ 30.9; *n* = 18	46.3 $\pm$ 31.0; *n* = 162	40.7 $\pm$ 34.6; *n* = 27
Length of hospital Stay (d)	10.5 $\pm$ 6.7	13.1 $\pm$ 11.15	14.4 $\pm$ 10.1
Osteoporosis	4	45	5
Mortality	2	26	12

**Fig. 2:**
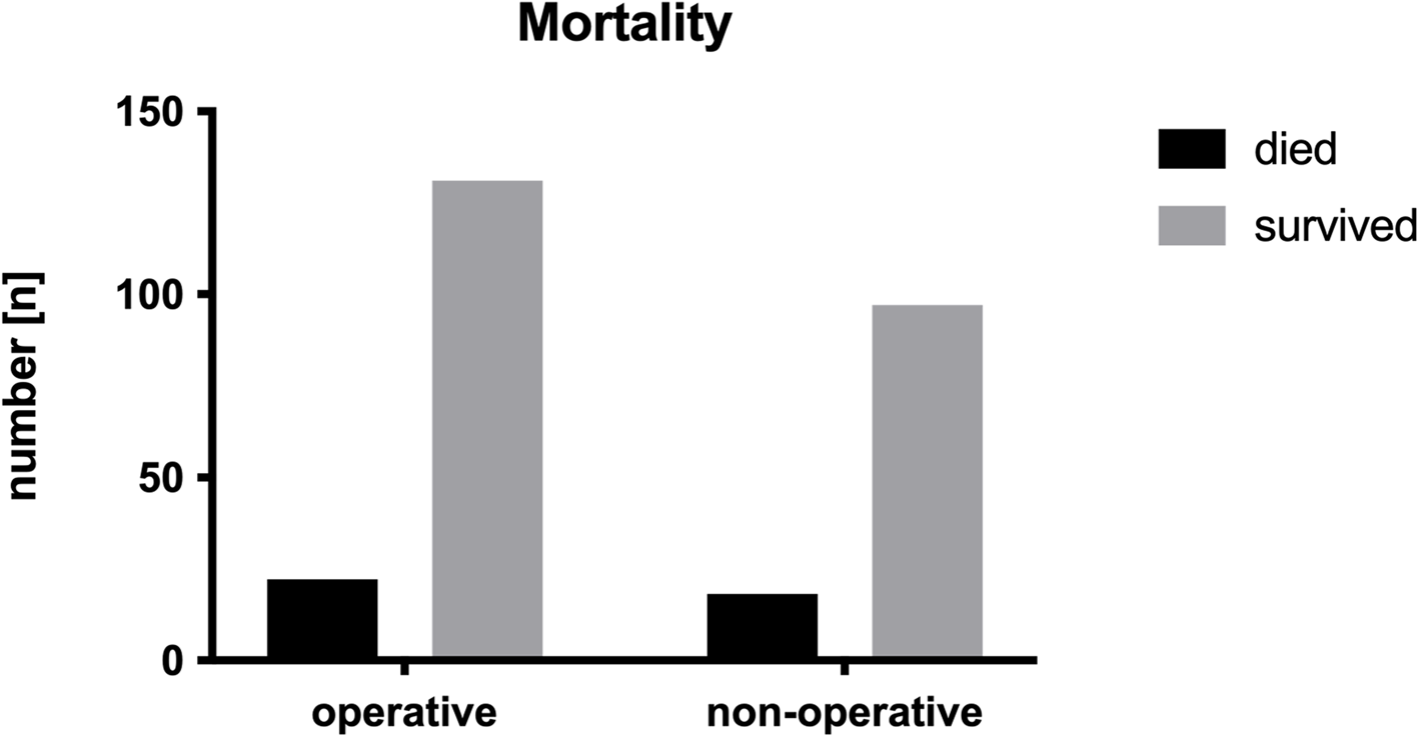
Mortality: surgical vs. nonsurgical: shows the number of patients who died and survived after surgery on the right and nonoperative treatment on the left

**Fig. 3:**
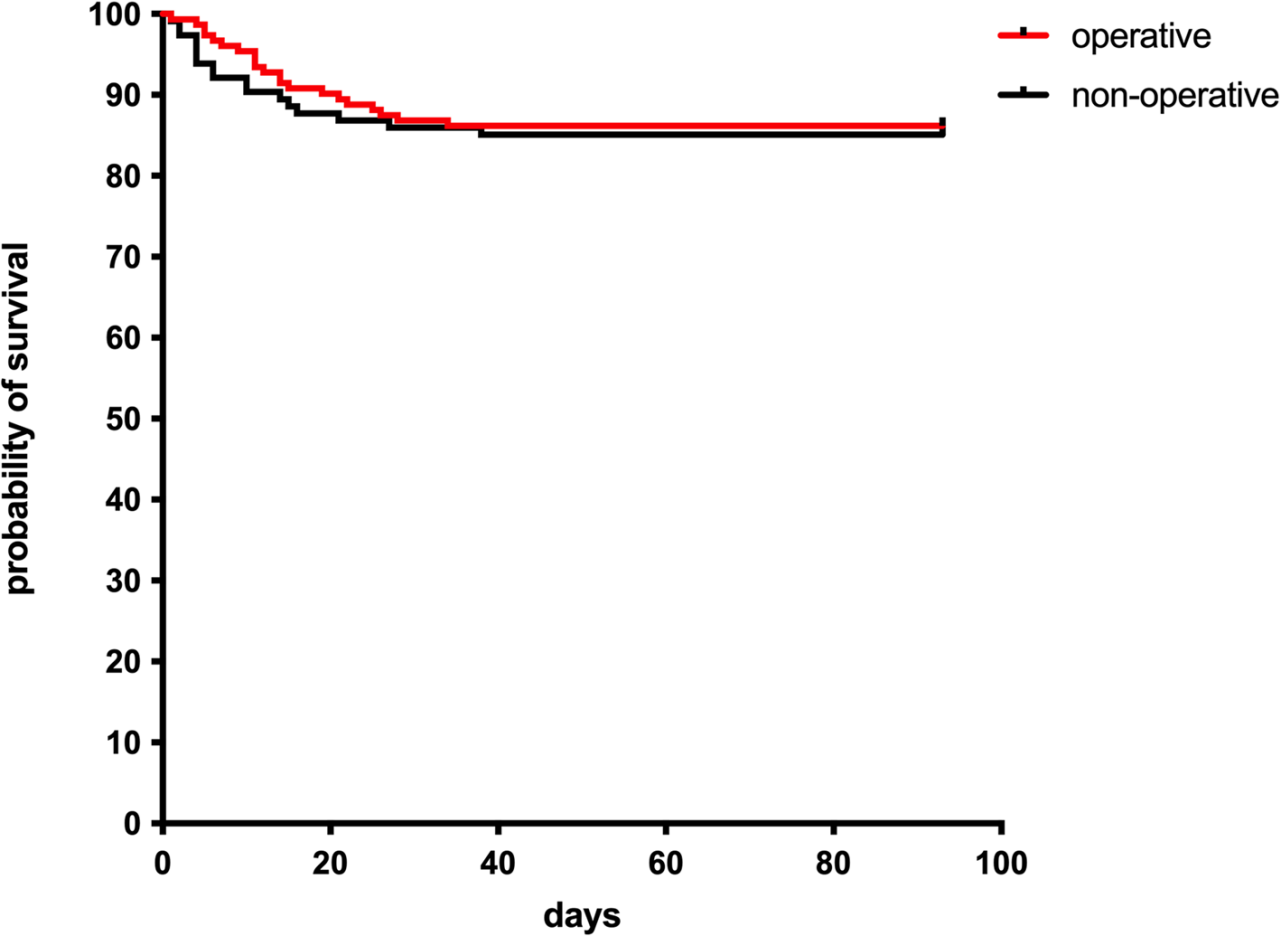
Kaplan‒Meier survival curve showing the probability of survival on the y-axis. On the x-axis are the days of survival comparing operative (red) and conservatively treated patients (black)

Comparing operatively and conservatively treated patients, independent of the fracture localizations, revealed no significant differences in mortality overall (*p* > 0.05; Fig. 2) and time-related (Fig. 3).

The differentiation of patients according to fracture localization and therapy is presented in Table 2.

**Table 2:** Shows the division of the fractures from Table 1 into operative and conservative groups by the same parameters but divided into the two groups that were considered separate in this study

		**C1**	**C2**	**C1/2**
**Conservative**	N	22	81	12
	Sex (m:f)	9:13	22:59	5:7
	Age	84.26.0	85.96.6	86.45.9
	ASA 3	14	60	11
	CCI	5.41.6	6.11.9	5.31.3
	Barthel (*n*=)	50.331.84; *n*=17	47.532.1; *n*=60	29.437.6; *n*=8
	Length of hospital stay	9.76.7	8.28.3	8.88.6
	Osteoporosis	4	18	2
	Mortality	1	11	6
**Surgery**	N	3	122	28
	Sex (m:f)	1:2	38:84	8:20
	Age	83.36.1	84.06.6	85.65.4
	ASA 3	3	102	28
	CCI	6.32.1	61.9	6.51.7
	Barthel (*n*=)	50; *n*=1	45.530.6; *n*=102	45.533.2; *n*=19
	Length of hospital stay	16.33.8	16.411.6	16.99.8
	Osteoporosis	0	27	3
	Mortality	1	15	6

Nonoperatively and operatively treated patients with a C2 fracture did not differ in CCI, Barthel, or the number of patients with an ASA ≥ 3 (*p* > 0.05). Nonoperatively treated patients with a C2 fracture tended to be older (*p* = 0.0563) and had a significantly shorter length of hospital stay (*p* < 0.0001). A shorter length of hospital stay (*p* < 0.0001) was also found for conservatively treated patients with a combined C1/2 fracture. Operatively treated patients with a combined C1-2 fracture had a significantly higher CCI (*p* = 0.0252) than nonoperatively treated patients. No significant difference in CCI was observed between the operative and nonoperative groups when considering odontoid injuries (*p* = 0.7257). There were no differences in age, Barthel Index or number of patients with an ASA ≥ 3 (*p* > 0.05). The overall mortality rate was 14.9% (*n* = 40) nonoperatively 15.7% (*n* = 18) vs. surgically 14.4% (*n* = 22) (*p* = 0.8628). There were no significant differences between nonoperatively and operatively treated patients regarding the diagnosis of osteoporosis and mortality for all fracture localizations (*p* > 0.05).

The complications are summarized in Table 3. The number of patients suffering from dyspnea was significantly higher in the operatively treated group (Table 3).

**Table 3:** Shows the rate of complications in conservatively and surgically treated patients, divided into general complications in both groups on the top with the p value on the right and, especially, surgical complications only in the group with surgical treatment. Every complication is shown with the absolute amount on the left and the relative amount on the right

	**Conservative (*n*** **= 115)**		**Surgery (*n*** **= 153)**		
	Absolute amount (n)	Relative Amount (%)	Absolute amount (n)	Relative Amount (%)	*p*-Value
Neurological Deficit	9	7,89	5	3,31	*p* = 0.1634
Delir	3	2,63	12	7,95	*p* = 0.1044
Reanimation	6	5,26	12	7,95	*p* = 0.4654
Impaired Mobilization	3	2,63	13	8,61	*p* = 0.0656
Dyspnoea	7	6,14	22	14,57	*p* = 0.0305
Sepsis	4	3,51	7	4,64	*p* = 0.7621
Urinary Tract Infection	9	7,89	21	13,91	*p* = 0.1701
Pneumonia	10	8,77	18	11,92	*p* = 0.4291
Bleeding	/		10	6,62	
Deep Wound Infection			5	3,31	
Malpositioning /Loosening			4	2,65	

The overall rate of general complications was 51.4% (*n* = 51) in the nonoperative group and 71.9% (*n* = 110) in the operatively treated group. The overall surgical complication rate was 12.42% (*n* = 19).

Of these patients, 21 had a C2 fracture, and one had a C1 fracture. In the C2 group, the surgical approach showed no significant differences (anterior, *n* = 18; posterior, *n* = 3; *p* = 0.1253). Preoperatively, four patients suffered from COPD, and one suffered from pulmonary fibrosis. The complications of the surgical procedures were summarized in Table 4. The in-hospital mortality rate for posterior fixation was 13.3% (6/45), compared to 12.3% (13/106) for anterior fixation. Statistical analysis revealed no significant difference (*p* = 0.82).

**Table 4:**
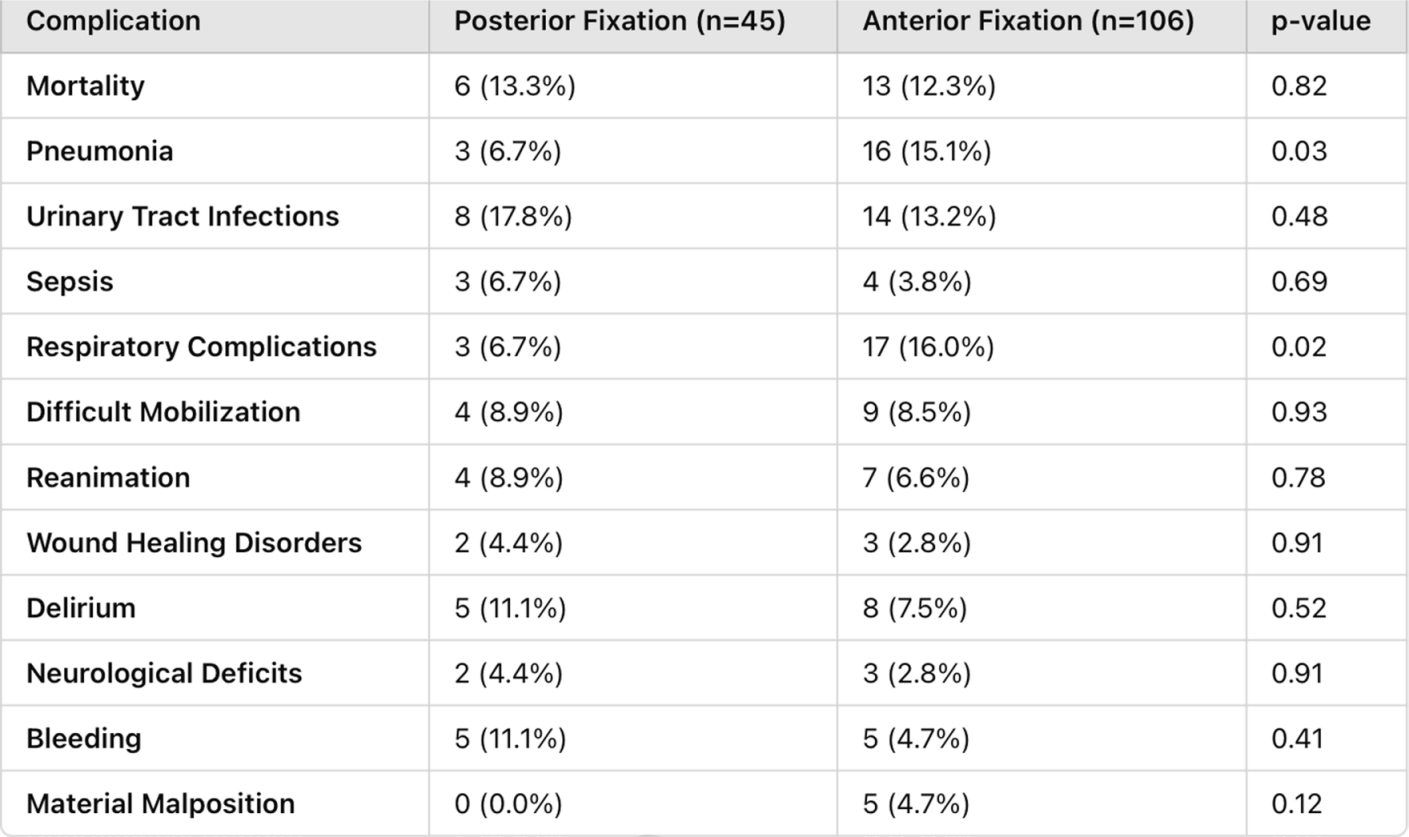
Summarizes the rates of complications and in-hospital mortality for posterior and anterior fixation groups

Pneumonia was significantly more frequent in the anterior fixation group (15.1%, 16/106) compared to the posterior fixation group (6.7%, 3/45) (*p* = 0.03). Similarly, respiratory complications occurred more often in the anterior fixation group (16.0%, 17/106) than in the posterior group (6.7%, 3/45) (*p* = 0.02). Rates of urinary tract infections (*p* = 0.48), sepsis (*p* = 0.69), difficult mobilization (*p* = 0.93), reanimation (*p* = 0.78), wound healing disorders (*p* = 0.91), delirium (*p* = 0.52), and neurological deficits (*p* = 0.91) did not differ significantly between the groups. Bleeding rates were higher in posterior fixation (11.1%, 5/45) compared to anterior fixation (4.7%, 5/106), but this difference was not statistically significant (*p* = 0.41). Material malposition was observed only in the anterior group (4.7%, 5/106), with a non-significant *p*-value (*p* = 0.12) (Table 4).

Among patients who underwent posterior occipitocervical fusion (C0-3 fixation), no postoperative swallowing dysfunction was documented. A thorough review of medical records revealed no cases of dysphagia requiring intervention. Given the small number of patients with occipitocervical fixation, a direct statistical comparison was not performed. However, no significant differences in overall postoperative complications or life prognosis were observed between patients with occipitocervical fixation and those without. Due to the small sample size of operatively treated C1 fractures, a statistical analysis was not performed.

**Fig. 4:**
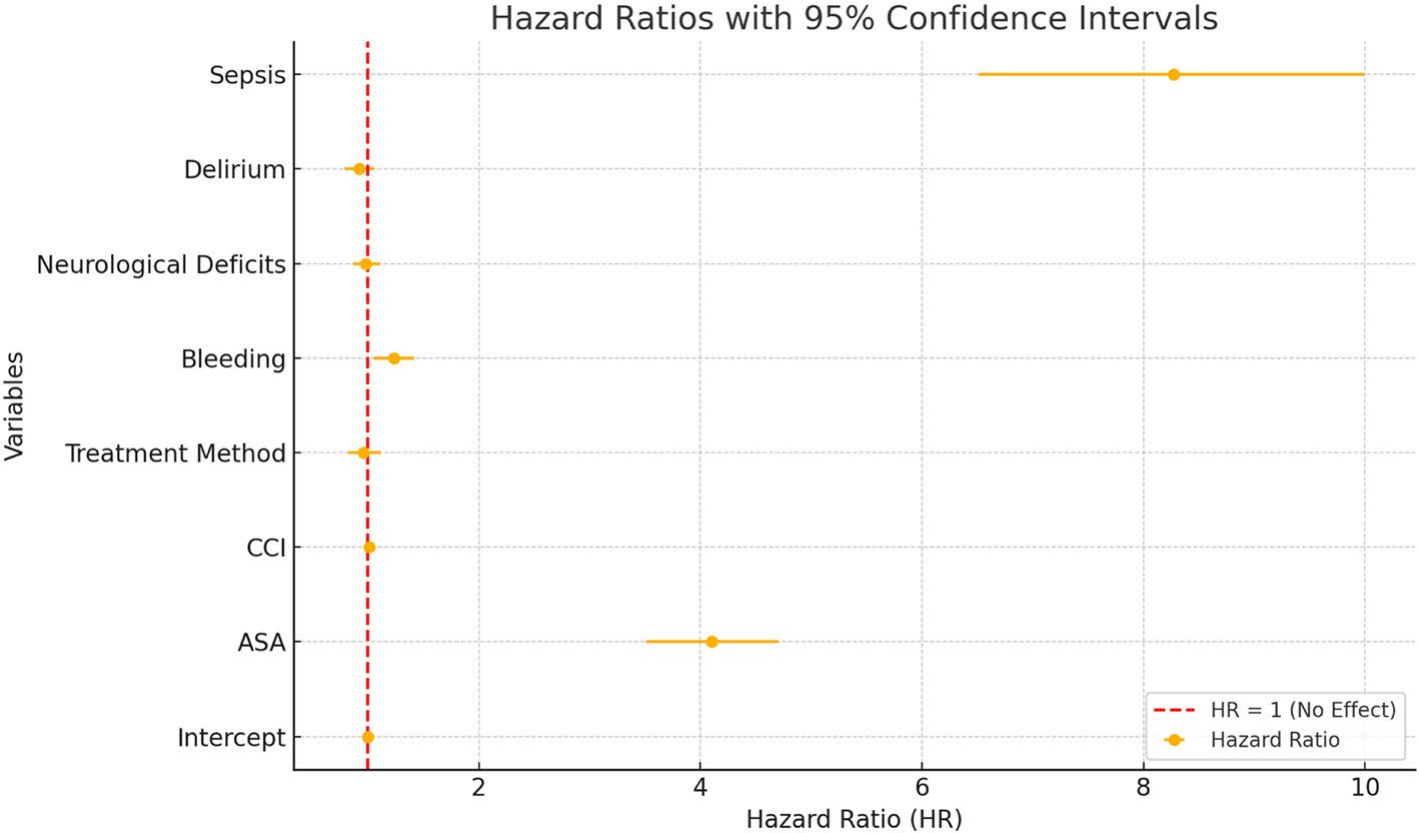
Hazard ratios with 95% confidence intervals for predictors of in-hospital mortality. Red dashed line at HR = 1 indicates no effect

The ASA score was identified as a significant predictor of in-hospital mortality, with a hazard ratio (HR) of 4.104 (*p* < 0.001), indicating an increased mortality risk with higher ASA scores. Among complications, sepsis was strongly associated with mortality, with a hazard ratio of 8.271 (*p* < 0.001). No significant difference in mortality was observed between operative and conservative treatment groups (HR = 0.960; *p* = 0.518). The Charlson Comorbidity Index (CCI) showed a weak association with mortality (HR = 1.013; *p* = 0.061). Operative treatment was linked to higher rates of respiratory complications, including dyspnea and pneumonia, particularly in anterior surgical approaches, without a significant impact on overall mortality (Fig. 4, Table 5).

**Table 5:**
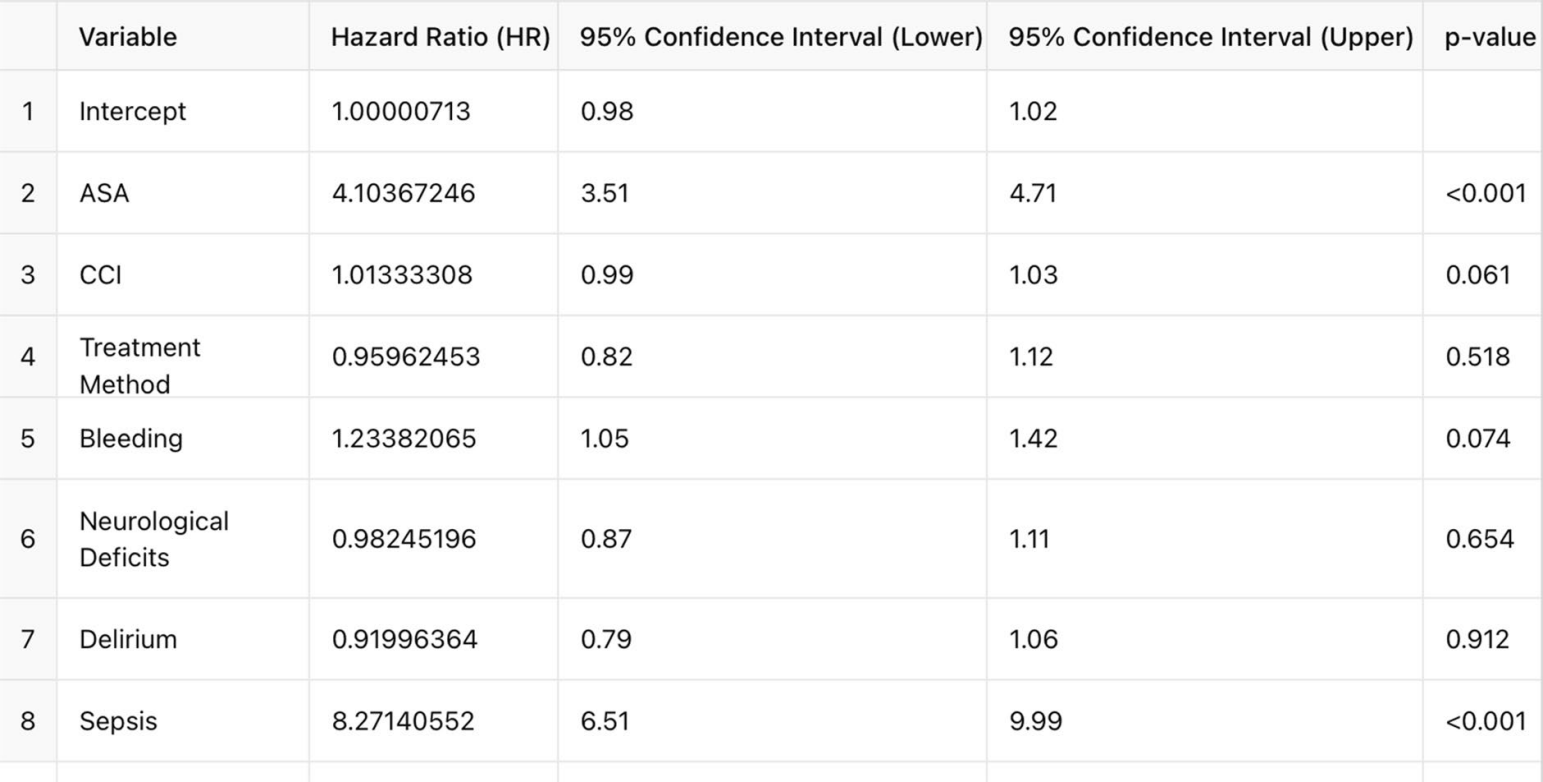
Hazard ratios (HRs) with corresponding 95% confidence intervals (CIs) and *p*-values for predictors of in-hospital mortality

## Discussion

Axial fractures are the most common injuries of the cervical spine in geriatric patients [19].

Degeneration in the atlanto-odontoid joint is found in 42% of patients aged 70 and 61% of patients aged 80 years, with an incidence of 90% from a median age of 79 years [13]. A variety of factors play a significant role in deciding on the treatment strategy, including osteoporosis, delayed healing, pseudarthrosis risk, pain and surgical risk increase as well as the risk of complications.

There are two basic approaches to the therapeutic option available. On the one hand, conservative therapy with the application of a soft collar brace versus surgical therapy via a dorsal or ventral approach is under discussion. While halo fixation is a recognized treatment for cervical spine fractures, it is often associated with high rates of complications, particularly in elderly patients. In our study, only one patient underwent halo fixation and experienced pneumonia as a complication. This aligns with findings by Muzumdar et al. (2006), who reported a 14% perioperative mortality rate in elderly patients treated with halo fixation, alongside complications such as respiratory distress, dysphagia, and pin-related issues [26]. The high complication rates may reflect significant underlying disease processes in this patient population.

The patients in our study had an average age of 84.9 years. Octogenarians or people over the age of 80 are at an increased risk for fractures due to age-related changes in bone strength and density [9, 10].

In a meta-analysis by Johnell and Kanis (2006), the risk of vertebral fractures was 14 times higher in women over 80 years of age than in women 50–59 years of age.

However, odontoid fractures have increased significantly over the last two decades. A Swedish registry study by Robinson et al. also showed that there is a trend that older patients are receiving less surgery than younger patients [27].

Whether surgically or conservatively treated, all patients had a certain baseline risk for longer recovery time than a younger patient and is expected to have more complications. which is reflected in the CCI and ASA.

In our study, the ASA score and sepsis were identified as the most significant predictors of in-hospital mortality in elderly patients with upper cervical spine fractures. A hazard ratio (HR) of 4.104 for the ASA score emphasizes its strong association with mortality, consistent with findings from previous studies, where ASA ≥ 3 has been shown to significantly increase perioperative risks [28]. Sepsis demonstrated an even stronger impact on mortality, with an HR of 8.271, aligning with literature that highlights the critical role of infection management in reducing mortality rates among geriatric patients [29].

Interestingly, the Charlson Comorbidity Index (CCI) showed only a weak association with mortality (HR = 1.013), suggesting that while comorbidities remain relevant, the ASA score may be a more robust predictor in this context. This aligns with prior studies indicating that the ASA score often outperforms CCI in predicting surgical outcomes, particularly in elderly populations with significant baseline risks.

Our findings revealed no significant difference in mortality between operative and conservative treatment groups (HR = 0.960), a result consistent with the literature [30]. Studies have shown that treatment modality itself may not be the primary determinant of survival but rather that outcomes depend on patient-specific factors such as overall health and comorbidities.

The higher rates of respiratory complications, including dyspnea and pneumonia, in operatively treated patients, particularly following anterior approaches, further emphasize the importance of individualized treatment strategies. Similar trends were observed in studies by Robinson et al. [27], which noted increased respiratory risks with anterior surgical approaches [27].

We found no significant differences in fragility fracture occurrence between men and women. However, women are at higher risk of vertebral fractures than men due to osteoporosis and postmenopausal changes. In a study involving 225 patients (113 males and 112 females) with an average age of 79.7 years (range 65–98), C2 type fractures were the most common (21.8%). The surgical group had higher rates of pneumonia and cardiac and respiratory failure. However, there was no significant difference in mortality between the groups [31].

In elderly individuals, White et al. showed that the most common complications were heart failure, thrombosis, stroke, pneumonia, respiratory failure, liver failure, and severe infections. In addition, it was shown that the rate of serious respiratory complications did not differ between anterior and posterior care, which is consistent with our results.

In our study, no significant differences in mortality were found between the surgically treated group and the nonoperatively treated group. Chapman et al. [15] reported a higher 30-day mortality rate in the nonoperatively treated group (*p* < 0.0001) of 7% (*n* = 11) operatively vs. 22% (*n* = 35) nonoperatively with a comorbidity rate (Charlson Comorbitity score of 1.1 + −1.5 operatively vs. 1.4 + −1.1 nonoperatively). In their population with 322 patients (mean age 81.8), the overall mortality was 14%.

Compared to this study, we found a sixfold higher CCI in our study population with C2 fractures (6.1 ± 1.9 in both groups).

There was a significant difference in CCI between the operative and nonoperative groups when considering C1/2 injuries (*p* = 0.0252). Patients from the surgical group appeared to be significantly sicker than the nonoperative patients.

The overall complication rate in our study was 51.4% (*n* = 51) in the conservative group. In the surgical group, the complication rate was 71%, and the most common complications were dyspnea, urinary tract infection, pneumonia and delirium. In contrast, surgery-related complications were found in 12.42% of cases. Osteoporosis and comorbidities are the main factors for the high complication rate in surgically treated patients, which may explain the lower length of hospital stay in the conservative group.

In a study by Yue et al. [6], 442 patients aged more than 80 years (octogenarians) who had been treated surgically for a C2 fracture were examined; the mortality rate was 9.7%, while the complication rate was 38.5%.

In a meta-analysis by Deng et al. [7] examining a total of 22 studies (case series and retrospective studies), the authors found that there was a higher complication rate in the surgical group (38.9%, 58/149; vs. 24.5%, 26/106), while no difference was found in mortality. In our group, there were no differences in the comorbidity rate (complication rate), except in dyspnea (*p* = 0.454), where there were significantly higher complications. The authors concluded that neither method showed any advantage surgically nor conservatively. Moreover, they pointed out that the limitation of their meta-analysis was the low quality of the available evidence [8]. In this regard, we looked at whether access morbidity plays a role and analysed whether the use of a dorsal or ventral approach may have an impact on dyspnoea but found no correlation.

Regarding surgical treatment procedures, our study identified significantly higher rates of pneumonia (15.1% vs. 6.7%, *p* = 0.03) and respiratory complications (16.0% vs. 6.7%, *p* = 0.02) in the anterior fixation group compared to posterior fixation. These findings are consistent with Boddapati et al. (2021), who reported respiratory compromise as a rare but severe complication of anterior cervical spine surgery, with an incidence of 0.57% in a large cohort. Risk factors identified included prolonged operative time, preoperative myelopathy, and multi-level procedures, which may explain the increased respiratory risks associated with anterior approaches [32].

Similarly, Cheung and Luk [33] highlighted that both anterior and posterior approaches present specific risks, emphasizing the importance of tailoring surgical decisions to the patient’s pathology and overall health [33]. While anterior fixation remains essential for certain indications, our findings suggest that posterior fixation may be a safer option for elderly patients at elevated risk of respiratory complications.

However, a French multicenter study examined conservative and surgical treatment of odontoid fractures in patients and found a significant overall mortality rate in the group older than 70 years of 16% within 1 year of observation [34]. There are several risk factors in the literature that increase mortality. A study by Ryang et al. [35] found that patients with multiple comorbidities had a higher mortality risk than patients without comorbidities. Additionally, delays in diagnosis and treatment may increase mortality risk. A study by Chibbaro et al. [36] found that a higher CCI is statistically associated with a higher nonunion rate. Even with a complication rate of 11%, a study by Ishak et al. [37] concluded that surgery should be considered the first-choice treatment. A study by Platzer et al. [38] found that delayed timing of surgery may increase the risk of postoperative complications. Moreover, the severity and type of fracture may also influence mortality risk. A study by Sheikh al [39] found a higher mortality rate in patients with severe odontoid fractures than in patients with less severe fractures.

We found no significant differences in gender. However, women have a higher risk of vertebral fractures than men due to osteoporosis and hormonal changes. According to a study by Kaesmacher et al., osteoporosis is the most important risk factor for odontoid fractures in elderly individuals [40].

Furthermore, with the geriatric patient population, it should be considered that geriatric adapted departments should be established. Furthermore, a basis for discussion should be created regarding whether older patients should be connected to geriatric facilities after discharge. Management by orthogeriatric care models may be particularly beneficial for older patients with fractures of the dens axis, atlas, and C1/2.

According to the literature, there are studies on hip fractures in which the implementation of orthogeriatric care models has improved outcomes in terms of in-hospital mortality, 1-year mortality and length of stay [41]. Geriatric centers can potentially improve outcomes by providing tailored care that addresses comorbidities, fall prevention, and rehabilitation. Standardized protocols and interdisciplinary collaboration may enhance the treatment of dens axis, atlas, and C1/2 fractures. Van Heghe et al. suggested that geriatric-focused care could significantly reduce mortality and morbidity in elderly patients [26].

### Limitations

This study has several limitations inherent to its retrospective design, which impacts the interpretation of our findings. As this is a retrospective study, treatment selection bias is an inherent limitation. The decision for conservative versus surgical management was not randomized and may have been influenced by factors such as fracture characteristics, surgeon experience, and institutional treatment protocols. Moreover, the choice of treatment (anterior vs. posterior fixation) was not randomized and may have been influenced by surgeon preference, patient comorbidities, or fracture characteristics. These biases may have led to systematic differences between treatment groups that are unrelated to the surgical approach itself.

Additionally, the retrospective nature limits our ability to control for unmeasured confounders, such as differences in perioperative care, rehabilitation protocols, or surgeon expertise, all of which could influence outcomes. Information bias is also a concern, as this study relies on pre-existing medical records, which may vary in quality and completeness. Missing or inconsistently documented data could obscure true associations or introduce inaccuracies in the reported complication rates. As osteoporosis diagnosis was based on existing medical records and not on systematic DXA screening, the true prevalence of osteoporosis in our cohort may be underestimated. Given the high reported prevalence of osteoporosis in octogenarians, this limitation should be considered when interpreting our findings.

Lastly, retrospective studies inherently lack the ability to establish causal relationships. While our statistical adjustments account for key covariates, such as age and comorbidities, residual confounding cannot be excluded. These limitations highlight the need for prospective or randomized studies to confirm our findings and provide a clearer understanding of the comparative risks and benefits of anterior versus posterior fixation in elderly patients with cervical spine fractures."

## Conclusion

The management of elderly patients with injuries of the 1st and 2nd cervical vertebra is a demographic challenge with a growing incidence. We demonstrated that in-hospital mortality and morbidity are high but did not differ between operatively and nonoperatively treated patients. The CCI and Barthel show the measure of the complexity of the patient population, which from our point of view needs peri- and postinpatient geriatric complex treatment. If possible, this should be adequately considered and, if necessary, cared for in the inpatient setting and underscores the necessity of establishing geriatric-specialized care structures.

## Data Availability

The datasets used and/or analysed during the current study available from the corresponding author on reasonable request.

## Abbreviations

ASA: American Sociation of Anasthesiology
CCI: Charlson Comorbidity Index
SD: Standard Deviation
COPD: Chronic obstructive pulmonary disease
